# A Rare but Reversible Cause of Lithium-Induced Bradycardia

**DOI:** 10.7759/cureus.8600

**Published:** 2020-06-13

**Authors:** Basma Ataallah, Rana Al-Zakhari, Aman Sharma, Michael Tofano, Gregory Haggerty

**Affiliations:** 1 Internal Medicine, Zucker School of Medicine at Mather, Port Jefferson, USA; 2 Internal Medicine, Northwell Health Mather Hospital, Port Jefferson, USA; 3 Internal Medicine, Richmond University Medical Center, Staten Island, USA; 4 Medicine/Cardiology, Northwell Health Mather Hospital, Port Jefferson, USA; 5 Graduate Medical Education, Northwell Health Mather Hospital, Port Jefferson, USA

**Keywords:** voltage-gated sodium channels, electrocardiogram, lithium, pacemaker, lithium-induced bradycardia

## Abstract

Lithium is a well-known medication that has been used for many years to treat mood disorders. One of its side effects is cardiotoxicity, which usually occurs at serum lithium levels > 1.5 mEq/L but rarely occurs when therapeutic levels of lithium are used. Other causes of bradycardia should be eliminated by performing a detailed workup that includes calcium level, thyroid function, and cardiac workup, with consideration of any medication interactions. Lithium-induced bradycardia is reversible upon discontinuation of lithium, but irreversible sinus node can occur and may warrant permanent insertion of a pacemaker to maintain sinus rhythm when long-term lithium therapy is required. Herein, we describe the case of a 42-year-old woman who presented with symptomatic bradycardia. Bipolar disorder was described in her past medical history, and she was receiving lithium therapy. A detailed workup indicated bradycardia secondary to lithium use. Her condition improved after discontinuation of the lithium, and normal sinus rhythm was restored over the next three days.

## Introduction

Lithium is efficacious for treating psychiatric illness, including mania and major depression associated with bipolar illness. However, the use of lithium at therapeutic and toxic serum levels is also associated with major cardiovascular side effects, including sinus node dysfunction ranging from benign to severe. Discontinuing lithium is the key to improving bradycardia in these patients, although some patients will require a permanent pacemaker to decrease symptoms upon resumption of lithium therapy [1].

## Case presentation

A 42-year-old woman came to the emergency department with concerns of dizziness and feeling sluggish for three days. One month earlier, she was hospitalized for the same symptoms and was discharged after discontinuing lithium. However, she experienced worsening manic symptoms, and it was recommended that lithium therapy be continued by her psychiatrist. Her medical history includes fibromyalgia and polysubstance abuse, including opioids (last use was 11 years prior to presentation) and crack cocaine (last use was one month prior to presentation). Additionally, her history includes the use of medical marijuana for pain management. 

**Figure 1:**
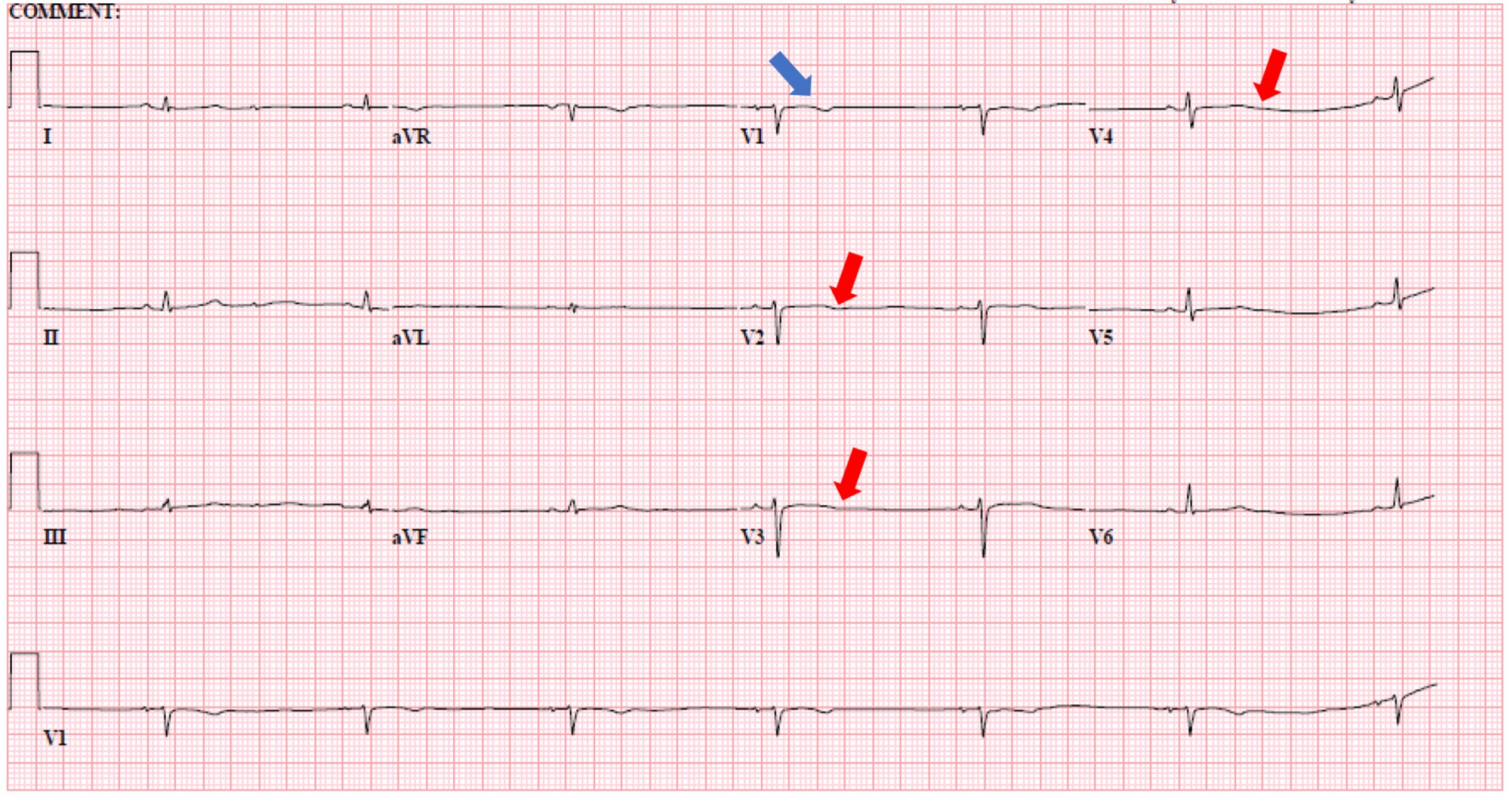
Electrocardiogram showing sinus bradycardia (rate of 41 beats/minute) with T-wave inversion in V1 (blue arrow) and T-wave flattening in V2-V3-V4 (red arrows).

On examination, she was found to have a heart rate of 41 beats/minute and a blood pressure of 91/35 mmHg. Her urine toxicology screen was positive for cannabinoids. Her electrocardiogram (ECG) was suggestive of sinus bradycardia (Figure 1), which showed normal left ventricular function (ejection fraction: 60%). Serum electrolytes, including calcium level and thyroid-stimulating hormone levels, were within reference limits. Treatment history revealed that her lithium therapy consisted of 300 mg three times per day, which was increased two weeks ago due to worsening mania. She had no personal or family history of heart disease, and no other medications had been prescribed that were known to cause heart block. Her serum lithium level (0.55 mmol/l) was within the therapeutic level. 

The bradycardia was thought to be due to lithium-induced sinus node dysfunction or idiopathic sick sinus syndrome (SSS). SSS was excluded because the heart rate increased to 65 beats/minute after discontinuing lithium therapy. Based on her drug history and investigations, the patient was diagnosed with lithium-induced sinus node dysfunction. Lithium, with a half-life of 18 to 36 hours, was discontinued, and sinus node function improved over the next three days. She was then treated with aripiprazole 5 mg once per day and discharged after five days. A pacemaker was not necessary because restoration to sinus rhythm occurred following the discontinuation of lithium.

## Discussion

Lithium was introduced 55 years ago as a treatment for psychiatric disorders, and it is currently a medication used to treat mood disorders. Given the long history of lithium’s use, it has been extensively studied, and its mechanism of action and long-term side effects are well known and documented. Cardiac side effects, including benign ECG changes to near-fatal arrhythmia caused by the use of lithium at therapeutic and toxic levels, have also been described in the literature. Despite the previous studies that documented lithium-induced bradycardia as a sequela of lithium-induced hypercalcemia and hypothyroidism, there have been few case reports examining sinus node dysfunction in patients taking lithium who were euthyroid and eucalcemic [1]. 

At present, the underlying mechanism of lithium-induced cardiotoxicity is not entirely understood. However, in one study, it was mentioned that lithium causes a dose-dependent blockage of myocyte voltage-gated sodium channels. It is well known that these channels play a major role in controlling the myocardial conductive current as well as the sinus nodal pacemaker activity. Because of this blockage effect, the intracellular potassium decreases, which causes an electrical instability in the atria and ventricles that leads to various electrophysiological changes, including a decrease in the depolarization rate and electrical impulse propagation. Another study mentioned that the sinus node could also be affected by lithium through its effect on pacemaker channels (hyperpolarization-activated cyclic nucleotide-gated [HCN] channels), L-type calcium channels, acetylcholine-gated potassium channels, and the sodium-calcium exchanger that controls the sinus node function through complex interactions [1,2].

Lithium-induced cardiotoxicity has been established at serum lithium levels >1.5 mEq/L, because high serum lithium concentration can lead to progressive disequilibria between the extracellular and intracellular sodium concentrations [2]. With the use of therapeutic lithium levels, one of the most common ECG findings is sinus node dysfunction and T-wave abnormalities. Flattening or occasional inversion of the T-waves is seen in 16% to 33% of lithium-treated patients, but this finding is reversible within two weeks after discontinuation of lithium and was also noted for our patient [2]. These changes rarely occur at therapeutic levels (0.6-1.2 mEq/L), leading to ECG changes [3]. 

Although lithium-induced bradycardia at therapeutic levels is rare and not extensively documented, a thorough workup should be performed for patients undergoing lithium therapy to rule out lithium as a possible cause. Monitoring hydration status, renal function, and salt balance in individuals receiving lithium therapy is recommended. Lithium should be discontinued if rhythm disturbances occur during treatment. When long-term lithium therapy is necessary for patients with bradycardia, cardiac pacing is an option that can maintain sinus rhythm so that maintenance treatment can be carried out [4]. Although reversal of lithium-induced sinus node dysfunction has been achieved following discontinuation of lithium, there have been occasional reports of irreversible sinus node dysfunction induced by re-challenge with lithium [4,5]. 

Lithium-induced sinus node dysfunction at therapeutic lithium levels is rare, and its true prevalence is still unknown [6]. Given that not all patients receiving lithium develop sinus node dysfunction, there may be other factors that affect conduction including fluctuating levels of serum lithium, intrinsic parasympathetic and sympathetic tone, age-related interstitial fibrosis, and decrease in sinus rate, variations in the expression of sodium channels, and underlying pre-existing cardiac disease [7].

The presentation of our patient with symptomatic bradycardia secondary to lithium use at the therapeutic level, and with normal kidney and thyroid function, and normal calcium levels is unique. The patient was managed by discontinuation of lithium without the need for a pacemaker or other aggressive measures.

## Conclusions

At the therapeutic level, one side effect of lithium is that it can cause sinus node dysfunction in patients with healthy thyroid function and calcium levels. The optimal diagnosis and management of such patients is the discontinuation of lithium and consideration of an alternative agent for underlying psychiatric problems. Although sinus node dysfunction is rare, careful follow-up monitoring is required. Patients should preferably avoid using lithium because of the possibility of recurrence of bradycardia and the potential for the irreversibility of the condition, as well as the subsequent need for a temporary or permanent pacemaker. 
